# Alternative Medicines on the Farm: A Study of Dairy Farmers' Experiences in France

**DOI:** 10.3389/fvets.2021.563957

**Published:** 2021-02-25

**Authors:** Florence Hellec, Claire Manoli, Manon de Joybert

**Affiliations:** ^1^ASTER, INRAE, Mirecourt, France; ^2^URSE, ESA, Angers, France; ^3^BIOEPAR, INRAE, ONIRIS, Nantes, France

**Keywords:** homeopathy, essential oils, holistic approach, animal health management, comprehensive investigation

## Abstract

Despite being of debatable efficacy, alternative medicines are in regular use on both organic and conventional dairy farms as part of a strategy for limiting the on-farm use of antibiotics. The study presented here examined French dairy farmers' understanding of and experiences with these medicines, focusing on homeopathy, aromatherapy and phytotherapy. Adopting an interdisciplinary approach combining animal science and sociology, we considered how dairy farmers' use of alternative medicines fits into a holistic approach to herd health management, on the one hand, and into farmers' networks of professional relationships, on the other. Our findings show that farmers are interested in alternative medicines for reasons that are at once technical, ethical, and economic. In the absence of local veterinarians specializing in homeopathy and aromatherapy, farmers enroll in short-term training courses to learn how to use these medicines. Alternative medicines are not a substitute for conventional medicine for these farmers; rather, they constitute one part of a holistic approach to herd health that combines preventive measures with a variety of curative treatments, and which is grounded in close attention to the animals' state of health. Farmers make use of guidelines for observing livestock that are central to the veterinary alternative medicine approach. Interestingly, women farmers appear to play an important role in introducing these practices into the management of the farm operation. Finally, farmers' interest in alternative medicines is indicative of their broader expectations for advice and support in moving toward the integrated management of livestock health, a key element of the agroecological transition. Recognizing these expectations offers useful insights for rethinking the role of veterinarians in dairy farming.

## Introduction

Stricter regulations for the use of antibiotics in livestock production were introduced in Europe in 2015 to help limit the spread of antimicrobial resistance (AMR) and preserve key antimicrobials for use in human medicine. The objective is to move toward a more prudent use of antibiotics for animal health on farm. In the bovine dairy sector, researches have shown that there is still inappropriate use of antibiotics, especially for udder health management (1, 2). Better adherence to treatment doses and durations, treatment thresholds or less systematic use of antibiotic therapy, especially for the drying up of dairy cows, are ways to reduce the amount of antibiotics used (3). Some scientific studies focused on the behavior of farmers with regard of antimicrobial use (AMU) in order to understand the reasons why they do not follow the general recommendations even when they are aware of them. These studies highlight the importance of local standards amongst farmers (4), as well as the economic and working time constraints that farmers face (5). AMU on farm is also directly influenced by attitudes of advisors, especially veterinarians, and regulations (6). In general, veterinarians, who are the main prescribers of antibiotics in animal husbandry, are considered key stakeholders in accompanying farmers to move toward prudent use of antibiotics (7, 8). Various studies suggest ways to improve collaboration between farmers and veterinarians on the issue of AMR (9, 10).

Another strategy to reduce AMU that is suggested by public policies is the promotion and the development of alternatives treatments. However, the notion of “alternative” is poorly defined and remains vague. In the French action plans that have been implemented to fight against AMR, the term “alternative” covers a wide variety of products and methods: preventive products such as vaccines, food supplements (probiotics), treatment products other than antibiotics such as macrophages or herbal products (phyto- and aroma-therapy) (11, 12)^1^. The use of “alternatives” is also considered by agricultural stakeholders as a way to limit AMU for animal health management. Studies conducted on farms engaged in AMU reduction trajectories show that the farmers commonly use phytotherapy and aromatherapy, but also homeopathy (3, 13). Although no general statistics on the subject exist, a variety of local surveys point to the importance of this phenomenon. In France, for example, according to Le Guénic (14), 19% of conventional livestock farmers and 72% of organic livestock farmers in the region of Brittany were using homeopathy and/or aromatherapy. According to a recent European study, homeopathy is regularly used on organic farms in Germany, Switzerland, Norway, and Greece (15). As these figures suggest, it is in the organic farming sector that alternative medicines are most widely used. Indeed, alternative medicines are cited in organic certification regulations as preferable to antibiotics, provided their efficacy has been shown (Council Regulation (EC) n°837/2007).

The question of the effectiveness of alternative medicines has been a focus of intense debate, however, both in the scientific community and among professional veterinarians. Veterinary homeopathy^2^ is among the most controversial areas, since clinical research trials have so far failed to definitively establish its effectiveness. For some, this is proof of homeopathy's lack of value (16); others argue that more research should be done (17).

The terms of the debate are different for phytotherapy and aromatherapy,^3^ both of which are based on traditional knowledge. Ethnobotanical studies have inventoried the different types of plants used by farmers to treat their livestock (18, 19). Here too, however, more research is needed, since the therapeutic properties of many plant species have not been determined scientifically. Questions have also been raised concerning the potential toxicity of some plant-based medicines, both for the animals being treated and for the human consumers of livestock products (20).

Given the lack of scientific information as to the risks and benefits of alternative forms of veterinary medicine, how can we understand farmers' interest in these practices? Why and how do farmers adopt these treatment methods, despite the fact that such methods are rarely recommended by veterinarians (21)? Do such treatments really constitute viable alternatives to antibiotic use, or do they in fact have no effect on animal health? Although some research (22, 23) has found that the use of alternative medicines does not add to farmers' management costs, this is not enough to conclude that they are effective.

The research described here sought to examine dairy farmers' uses of alternative medicines as a way of better understanding their interest in these treatment methods. We focused on homeopathy, aromatherapy, and herbal medicine, since these are the types of alternative medicine most widely used in France.^4^

Our goal was to move beyond an exclusive focus on farmers' motivations—an approach that has previously been explored [(23, 24)]—and toward a more detailed understanding of the livestock management practices associated with the use of alternative medicines to treat animals on the farm. To do so, we needed to reposition these practices within farmers' socio-technical and socio-professional systems. We thus adopted an interdisciplinary approach, combining a livestock farming systems perspective (25) with a sociological framework (26) to examine how the use of alternative medicines fits into farmers' overall management of herd health, on the one hand, and into their networks of professional relationships, on the other.

## Materials and Methods

This article is based on three distinct datasets from three different field studies conducted in different parts of France (Franche-Comté, Normandy and the Grand Ouest), within the framework of separate research projects. The methods of data gathering and analysis were qualitative: the goal of this article is not to provide a quantitative account of the phenomenon under investigation, but instead to assess its variations (27). Compiling data gathered within the context of three distinct research projects in different regions enabled us to review a wide range of uses of alternative medicines by dairy farmers in France.

The first two datasets come from ethnographic research conducted in Franche-Comté in 2016 (COPPECS project) and in Normandy in 2017 (Normandy region project) (In the remainder of this article, we will refer to these as Study FC and Study N, respectively). Our methods consisted of comprehensive interviews with farmers and with the instructors of farmer-training courses, in addition to phases of participant observation. Our objective was to study how breeders use alternative approaches of animal health on their own herd.^5^

For Study FC, we conducted eight comprehensive interviews: six with individual dairy farmers in Franche-Comté who had completed one or more training courses in animal health; one with a facilitator at an agricultural training center; and one with a veterinarian who offers homeopathy trainings. We identified interviewees with the help of intermediary organizations (not specific to organic farming) that offer short courses for farmers in animal health. We selected the farmers to be interviewed from the lists of participants in these training courses. This strategy enabled us to identify dairy farmers who regularly use homeopathy and/or herbal medicine and aromatherapy to treat their animals. We also participated in a group discussion day for dairy farmers on the topic of herd health management. Study N was conducted in collaboration with an agency providing technical advice and support for farmers that was seeking to improve its services relating to animal health. Over the course of one year, we followed a group of six dairy farmers (men and women) who were meeting regularly with an advisor to discuss holistic approaches to herd health. Having originally met through training courses in aromatherapy, these farmers wanted to learn more about other alternative methods for livestock health and to engage in peer-to-peer discussions about herd health management. We attended three meetings of the group during the year and conducted individual interviews with two of the farmers.

In these two geographic study areas, the interviews we conducted with the farmers were sociotechnical in nature; that is, we were interested both in the farms' herd health practices and in the farmers' understandings and descriptions of animal health. The interviews also included a section on the farmers' connections with their socio-professional environment.

We constructed an interview guide listing the different topics to be covered in our interviews with the farmers. Questions were open-ended, giving the farmer the opportunity to speak at greater length on the subjects he or she felt were important as well as to raise new topics we had not initially asked about.^6^ These interviews were recorded, transcribed in full, and then subjected to a content analysis using the Grounded Theory method (28), with the goal of understanding the farmers' perspectives on their experience. Following an inductive process, and using the farmers' practices and points of view as a starting point, we constructed an analytical grid highlighting the key themes that emerged from the interviews.

The analytical grid was developed in several steps. First, we defined broad themes based on the elements we had sought to examine; for each theme, we identified the relevant sections of the interviews. Next, within each broad theme, we identified a number of sub-themes based on the interview material. Here we followed an iterative process, going back and forth between the interview material and the analytical grid, eventually arriving at a stabilization of how the themes and sub-themes were designated. For example, in the initial grid we designated homeopathic practices, aromatherapy practices, and phytotherapy practices as three broad themes. In re-reading the interviews, however, we observed similarities in the approaches to livestock health management associated with these different types of medicines, including the central importance given to observation of the animals. We therefore decided to designate a broad theme relating to animal observation and then to distinguish sub-themes for the types of medicine in order to identify the variations in observational techniques associated with homeopathy, aromatherapy, and phytotherapy.

In the Grounded Theory method, the process of data interpretation is achieved through the construction of the analytical grid. The research results presented in the following section are therefore organized according to the themes and sub-themes defined in the final phase of the analysis. Studies FC and N enabled us to analyze how farmers access these forms of alternative medicine through training opportunities, and how they then implement the techniques they have learned on their farms. The interviews conducted with the other actors (group leader, veterinarian) and the observations of group situations enabled us to better understand the social context within which familiarity with alternative medicines is acquired by dairy farmers.

This analytical grid was then put to the test of analyzing the data from the third set of interviews, conducted in 2015 in the Grand Ouest^7^ (Brittany and the Pays de la Loire) as part of a study of the use of homeopathy on organic dairy farms (European project IMPRO). We will call this Study GO.

Study GO included 15 interviews with bovine dairy farmers who had converted to organic production and who were using or had used homeopathy to treat their herds. Interviews were conducted either with or without the farmers' veterinarian present. Interviewees were identified with the help of agricultural advisors and technicians specializing in organic farming, or through rural veterinarians. These interviews sought to examine farmers' practices in veterinary homeopathy and their views about this type of medicine more broadly. The first part of the interview took the form of a questionnaire^8^; the remainder was more open-ended, inviting the farmer to express why he or she became interested in homeopathy and the role homeopathy plays in the farm's overall management of herd health. The presence of the veterinarians did not constitute an impediment to free expression on the part of the farmers. On the contrary, we observed that the farmers did not hesitate to speak openly about their practices with regard to homeopathy, even when the veterinarians considered those practices out of line with their recommendations. In addition, the farmers defended their views with regard to the advantages and disadvantages of homeopathy, notably in situations where they disagreed with the veterinarians.

The semi-directive portions of the interviews were recorded and transcribed in full. These sections were then analyzed using the analytical grid developed for the first two datasets. The analytical grid proved to be robust: the themes and sub-themes did not need to be modified. We were thus able to enrich and refine the results by adding these interviews with organic dairy farmers, whose experiences with veterinary homeopathy is notably different from that of non-organic dairy farmers. Data saturation was achieved by combining and comparing the results of these three studies.

The analytical grid that emerged from this work featured the following themes:

- Why the farmers became interested in alternative medicines; and what steps they took to learn more about these different approaches and treatments
- The most important herd health issues on the farm; methods used to address these issues; any recent changes in this regard
- The different alternative medicines used on the herd; for which kinds of health problems; and the advantages and limitations of these methods
- Any other management changes made on the farm to address herd health
- Use of local veterinarians and/or other advisors on livestock health

In addition, we gathered factual data on the basic characteristics of the farms and their production systems, including herd size, number of employees, and participation in various quality schemes (organic, geographical indications, etc.).

## Results

### Study Sample

The combined study sample (made up of data from Studies FC, N and GO) consisted of 23 farms specializing in milk production, the large majority of which (21) were certified organic. Four farms were producing milk for Comté, a cheese that enjoys PDO (protected designation of origin) status and is subject to specific production requirements and quality specifications. One farm was both organic and a milk producer for Comté. Only two farms, both located in Normandy, were not enrolled in a quality-assurance certification program.

Herd size ranged from 37 to 130 milking cows (average herd size in France is 59 milking cows). Breeds of cows varied considerably: only two farmers, both of them organic and both located in the Grand Ouest, had herds made up exclusively of the breed known as Prim-Holstein (the French sub-type of the Holstein breed), the leading dairy breed in France. The farmers in Franche-Comté and in Normandy were all milking the breeds specific to their respective regions, Montbéliarde in Franche-Comté and Normande in Normandy. The remainder of the organic dairy farmers interviewed had herds made up of various breeds, including mixed-breed animals in some cases.

The number of employees per farm ranged from one to four man-work units (MWU)^9^ with an average of 2.35 MWU (the French national average for dairy operations was 2.18 MWU in 2016) (29). Eleven farms employed two people, usually a husband and wife owning and operating their farm together.

The individual(s) we met with at each farm were the person or persons primarily responsible for herd health. Eight interviews were conducted with a female farmer only (three in Franche-Comté, two in Normandy, and two in the Grand Ouest); ten with one or more male farmers (three in Franche-Comté and seven in the Grand Ouest); and five with a couple working together on the farm. We can see from these numbers that women farmers play a significant role in the management of herd health.

### A Different Approach to Care

The farmers we interviewed explained that their interest in alternative medicines emerged from a desire to take a different approach to livestock health—specifically, one that would be less reliant on antibiotics. For the organic farmers, certification rules require that they use antibiotics more sparingly; but both organic and non-organic farmers emphasized economic motivations for their interest in alternative medicines. Antibiotics and conventional veterinary medicine in general were described as very expensive. When antibiotics are administered to a milking cow, moreover, its milk has to be dumped for a period of several days, resulting in a loss of revenue. Farmers also underscored the high veterinary bills associated with a herd that is not in good overall health. One female farmer expressed the fear that some antibiotics currently used on dairy farms may be prohibited in the future.

In addition to these economic motivations, the farmers we interviewed emphasized concerns relating to their animals' welfare. Many said that they don't like administering injections. Adopting a different approach to veterinary care thus also meant choosing medicines that were less painful for the animals. Farmers' explanations frequently interwove economic motivations with ethical motivations, as is evident in the following exchange:

Researcher: What made you want to take these types of training courses?

*Farmer: Well, in the first place because it is less expensive. And then also because I'm convinced there are other ways to take care of the animals than… just giving them shots. We don't use any vaccines for our animals either. I don't believe in giving the animals injections all the time. There's no need for it*.

(Interview with a female farmer in the Jura, February 2016)

Farmers' interest in alternative medicine is thus inscribed within a larger questioning of how dairy animals should be cared for. For the farmers we interviewed, the goal was not simply to substitute one type of medicine for another, but rather to change their approach to animal health: placing more emphasis on limiting risks to animal health, interpreting those risks differently, thinking more about prevention. In Franche-Comté, the farmers we interviewed had taken a number of different training courses relating to animal health, including alternative methods (acupuncture, osteopathy) and preventive methods (feeding programs, ways of managing calves, etc.). In Normandy, the women farmers we met with had begun by taking courses in aromatherapy, but at the time of our study had also become interested in other alternative methods.

We also observed that this questioning of dairy management practices tended to emerge at a particular moment in a farmer's career—a point at which inherited or previously established farming practices could be reconsidered. Thus, for seven of the farmers we interviewed (five women and two men), pursuing training courses in alternative methods coincided with starting a farm, taking over the farm from a relative, returning to the farm after a period away, or otherwise making a new investment in a farm operation. In the interview excerpt below, for example, the (female) farmer started farming with her husband when her father-in-law retired. She decided to focus on animal health in part because she wanted to change the way her father-in-law had managed the calves:

Researcher: So, you had what you needed to start farming. Did you have to do some new training?

*Farmer: No, no, these were courses for a PPE*.^10^ *You do that for 5 years, it's required, but I found it interesting. And then I did a training course on calves because I realized I missed that, since my father-in-law was in charge of them before. When I arrived, he said to me: here, you take the baby (laughs). but the way he managed the calves'health… it didn't fit to me, I felt it was too abrupt, I had to find solutions to manage it differently*

(Interview with a farmer in the Jura, February 2016)

For some of the organic farmers we met with, a desire to change their approach to herd health helped prompt their decision to transition to organic, while for others, it emerged as a consequence of their shift to organic. For one part of organic farmers we interviewed, participation in training courses on alternative approaches to herd health predated by several years their decision to convert to organic: for them, organic agriculture was the realization of a longer-term commitment to changing their livestock management practices and in some cases their crop management practices as well.

*Farmer: In any case, when I started practicing homeopathy–it was long before we went organic, and there were no regulations then…. It was more an issue of animal health that got me into homeopathy… and animal welfare, more than anything else*.

(Interview with a dairy farmer in Ille-et-Vilaine, December 2014)

Other organic farmers first learned about alternative medicines during the transition period—often through participating in training courses and discussion groups with other organic dairy farmers, or other farmers transitioning to organic. Indeed, one of the major concerns for farmers converting to organic is finding therapeutic alternatives to the use of antibiotics, with the objective of complying with the requirements of the organic farming specifications.

### Farmer-Training Programs as an Entry Point for the Use of Alternative Medicines

Training courses relating to animal health, generally offered through agricultural organizations and agencies, were the principal route by which farmers learn how to use alternative medicines. Usually these take the form of short training courses, from one to several days in length, scheduled in the winter when farmers' workloads are relatively light. The farmers we met with in Franche-Comté all learned how to use alternative medicines for the management of herd health thanks to courses offered through the regional adult agricultural education agency. In Normandy, one of the women farmers we interviewed first learned about veterinary aromatherapy from an article in a professional agricultural journal. She tried using the techniques on her own, but without success. Then she saw a notice in the local agricultural newspaper that an alternative agriculture technical organization in her region was offering courses in veterinary aromatherapy. The other female farmer we interviewed in Normandy learned about these courses from a neighboring organic dairy farmer who had participated in the trainings previously. Of all the farmers in Study GO, only two did not receive any short training on alternative medicine. All the others famers interviewed in the Grand Ouest had learned about alternative medicine via training courses offered through professional organic dairy organizations. For the most part, the instructors for these courses are veterinarians specializing in homeopathy or phytotherapy who travel throughout France to teach courses of this type. Other professionals are sometimes also involved, including a psychologist trained in naturopathy who has developed his own approach to the use of aromatherapy to treat farm animals. As we saw when we participated in the training days, the instructors for these courses emphasize a holistic approach to animal health—their presentations are not limited to the use of alternative treatments based on homeopathic granules and essential oils. They also address broader topics such as how to properly observe an individual animal's condition, how to detect early signs of health problems, and how to use preventive methods to minimize health issues.

The central role of short training courses as an introduction to the use of alternative medicines stands in contrast with the weakness of other forms of advisory services and technical support for alternative veterinary medicines in the regions where we conducted our research. The farmers we interviewed typically only called their local veterinarians in emergencies or for the most serious health problems. They said they didn't speak to these vets about the alternative medicines they were using; or if so, only rarely. One farmer in the Grand Ouest, for example, lamented the lack of interest in alternative medicines among the veterinarians belonging to his local practice:

Researcher: Can you call a veterinarian to treat an animal using homeopathy?

*Farmer: No, unfortunately. Here, we have no one.… That's it; I can't look to my local vets for help! It's not even worth trying*.

(Interview with a farmer in Ille-et-Vilaine, December 2014)

A few farmers were able to call on the services of a homeopathic veterinarian located near their farms. This was the case for two male organic farmers in the Ouest and for one female farmer in Franche-Comté. The other farmers we met with had no access to a homeopathic vet in the rural veterinary practices in their area. For aromatherapy, no local advice was available for any of the farmers we interviewed.

Thus, as there is very little individual service offer in alternative medicine, short training courses are the main way for farmers to access to alternative medicine. Following the trainings courses, important connections can be forged between farmers and course instructors. Some farmers had taken several courses with the same instructor. Two levels of veterinary homeopathy, “introductory” and “advanced,” are typically offered for farmers. Among the aromatherapy group we followed in Normandy, the farmers met once a year with the instructor or with another individual trained in the instructor's approach. They used this time to review treatments that had worked for them, specific challenges they had encountered, or health issues they were contending with more generally. The instructor would ask the farmer about the symptoms they had observed, and could thus restate the key points to be observed in assessing an animal's health. The instructor could also review hygiene practices and dietary strategies to minimize health problems.

Another type of connection can also develop between famers and course instructors through the use of remote advisory services. Following training courses in veterinary homeopathy, one organic dairy farmer in the Grand Ouest and one dairy farmer in Franche-Comté stayed in touch with the veterinarian-instructor for individual advice. In the case of the Franche-Comté dairy farmer, these advice sessions were conducted with the veterinarian in person, on an annual visit to the farm—together, the farmer and the vet would review the herd's health and discuss additional preventive measures to put in place. For the two Grand Ouest dairy farmers, further advice from this veterinarian took the form of telephone conversations concerning a specific health issue with sick animals.

Farmer-to-farmer discussion groups are another means by which the connections established in training courses can be extended. We identified one such group in Franche-Comté (to which three of the female dairy farmers we interviewed belonged), focused on the topic of homeopathy. A male dairy farmer we met with in Maine-et-Loire belonged to another group focused on various aspects of herd health management, including homeopathy. The goal of these groups was to share successes and failures in herd health management, to improve farmers' observational skills with their animals, and to extend their knowledge of different homeopathic remedies. In these groups, farmers try to follow the “unicist” principle of homeopathy, according to which the remedy is determined by the animal's individual characteristics and the manner in which the disorder presents itself, not simply by the disorder itself or the underlying disease agent. The farmers keep notes on each animal they have treated using homeopathy, so as to have detailed, individualized account of herd health problems. These notes support in-depth discussion about famers' use of homeopathy. Generally speaking, however, the content of these farmer-to-farmer exchanges related to all aspects of herd health, not just the use of alternative treatments.

In this way, the training courses are the starting point for new relationships between breeders, and new collaborations between breeders and specialists in alternative medicine. These training courses are also a place of information on suppliers of herbal or homeopathic products. Most farmers buy the products that have been advised to them in training. Sales technicians that sell ready-to-use products made from dried plants or aromatic extracts can also advise farmers on their use. Some of the farmers we interviewed said they consult these individuals and purchase their products to address specific risks to herd health.

### Farmers' Uses of Alternative Medicines Are Diverse

We observed a wide variety of ways in which alternative medicines are used by dairy farmers to manage herd health. This diversity of practices is manifested first of all in differences in the level of understanding of these medicines: some farmers always used the same remedies for the same problems, while others sought to tailor each treatment to each case by closely studying the animal's condition.

These differences in approach in turn depended on the farmer's level of personal investment in learning about the techniques. Some farmers used the training courses to acquire a handful of “recipes” or simply purchase ready-made products: these might be mixtures of essential oils or homeopathic “compounds.” In the following interview passage, for example, the farmer explains how he uses several products containing essential oils to treat udder problems:

Researcher: And then you use herbal remedies… say for mastitis?

*Farmer: For mastitis. During the lactation*.

Researcher: And the product is… ?

*Farmer: There are two products… I mix them and then I put them down the cow's throat… It's from APA, it's an anti-infective… it's [the brand name] Gentiana*.

Researcher: So, it's an phytotherapy, is that right?

Farmer: Yes, and then the other one is… Arobactole? Let me look, I can't remember! Here, it's Symbiopole, I mix these two products and…

Researcher: Ok. And so you do that as soon as you notice… ?

Farmer: As soon as there is mastitis…

(Interview with a male farmer in Haute-Saône, February 2016)

Other dairy farmers take steps to further advance their expertise in alternative medicines. As we have seen, acquiring expertise in alternative medicines is a long process, typically involving multiple short training courses and, in many cases, participation in a discussion group.

The diversity of uses of alternative medicines is also manifested in the ways farmers combine different alternative medicines together and with conventional medicine. All of the farmers we interviewed used a variety of therapeutic approaches, either in parallel, for different types of health issues, or for a single type of health problem in the herd. All also continued to use antibiotics, although they reserved them for the most serious cases: antibiotics were either administered immediately for animals with the most serious symptoms, or kept as a backup strategy if a homeopathic or aromatherapy treatment proved ineffective. Some farmers also combined different types of alternative medicines. Among the 15 organic dairy farmers interviewed with respect to their use of homeopathy, for example, two also used aromatherapy, either at the same time as homeopathy, or for different types of problems among the herd.

For example, in the interview selection just cited, the farmer said he uses two herbal products to treat mastitis. He went on to say that about half the time, he also has to use an antibiotic:

Researcher: APA and Arobactole. The two together?

*(…) Farmer: 50 ml of APA and then 75, 70… yes, about 70… of the other*.

Researcher: Ok, got it. In the infected quarter?

*Farmer: No, no: for that, you grab them and put it down their throat. That one is an oral solution. You use the gun … And then if that doesn't work, then we go to Mastijet… we go to the antibiotic*.

Researcher: Ok. And does that happen often, that you have to use Mastijet?

*Farmer: We have very, very few…. that is, now we have almost no … It's the related factors: we have lower cell counts, so for mastitis, we probably have… maybe not even 20 per year*.

Researcher: Cases of mastitis where you use essential oils?

*Farmer: Cases where we use a treatment, yes. There are not even 20 per year… So you can put that there are 50%, I think that… for about 50% of the cows, it will work. For 50% we go to antibiotics*.

(Interview with a male organic farmer in Haute-Saône, February 2016)

In Franche-Comté, where the dairy farmers we met with regularly participated in short training courses on animal health, one farmer said he would call an osteopath for animals showing signs of lameness, or to check on a cow and calf following a difficult birth. One female farmer we met with practiced acupuncture in addition to using homeopathy, herbal remedies, and aromatherapy. In the passage given below, the researcher is asking the farmer about the treatments she uses for different health problems. She uses aromatherapy for mastitis, homeopathy for metritis, and in some cases both homeopathy and osteopathy for lameness:

Researcher: For example, for mastitis, is there a type of mastitis where you would normally use homeopathy and another type where you would do something else?

*Farmer: No. For any case of mastitis, I always use aromatherapy. For now, it works. But I don't have many, either… Once I had to bring in [the veterinarian] for a mastitis caused by a pathogenic E. coli and the animal was really in a bad way and at the time… Well, it was really at the beginning for me [using homeopathy] and I thought, I'm not going to tackle this on my own because she might not recover. So that was the one time where… she was lying down and… not doing well at all*.

Researcher: Metritis, in general, can that be treated with homeopathy?

*Farmer: Yes*.

Researcher: And lameness, do you have cases of lameness where you call the vet?

*Farmer: The osteopath*.

*Researcher: Oh, the osteopath*.

*Farmer: Yes. Because for lameness, I begin with… Well, in the first place, I don't always know if it is a paronychia, or if it's… So I always start with Pyrogenium. Right away, I see if that has an effect or not. Then there can also be lameness after calving, so then I would give Hypericum, or things that are more… you know, if I think there is a problem. I use Arnica after almost every calving. And then, well, you know, afterwards, if that hasn't worked, if I don't see that the hoof is swollen, all that, I call the osteopath. Yes. In fact we never have the vet come for lameness, really*.

(Interview with a female farmer in Mayenne, January 2015)

The use of different therapeutic approaches to animal care thus involves choosing among different types of possible assistance, corresponding to different methods: the local veterinarian is called in for emergencies and the most serious cases; specialists such as the osteopath may be called in for some specific types of problems; some farmers remain in contact with the veterinarian/course instructor for telephone consultations or occasional farm visits; while the farmer him- or herself administers some treatments, including alternative medicines, after having taken a few courses.

### Alternative Medicines in the Overall Management of the Dairy Operation and Herd Health

For many of the farmers we interviewed, animal health was a central preoccupation; alternative medicines were simply one tool among others within a holistic approach to herd health. Indeed, when we reposition the use of alternative medicines within overall herd management, we can see that dairy farmers use a variety of different measures to reduce health risks. Particular attention is paid to managing the cows' diet so as to limit health problems, especially metabolic disorders linked to milk production (acidosis, metabolic problems associated with calving, etc.). More broadly, we see that the overall improvement of herd health is linked to preventive measures that correspond to changes in livestock management: better management of feeding, particularly by adjusting the nutritional balance of the ration using the Obsalim® method^11^; and changes in housing to improve the animals' comfort and minimize unhealthy conditions.

At the same time, the farmers we interviewed emphasized that learning about alternative medicine had taught them how to observe their animals more closely and more precisely, and to identify signs of health problems they were unaware of before:

*Male farmer: In fact, I have learned… even if I am not… anyhow, I'm not going to brag about my skills in homeopathy! But… but still. I will say that for me the big advantage of the homeopathic approach is that I have learned to observe my cows. That's the most important thing for me! That's what homeopathy has done for me. At first, it's that… it's that*.

(Interview with a farmer in Maine et Loire, December 2014)

*Female farmer: What I've gotten from homeopathy, I often say, is how to make a diagnosis. That for me is… absolutely the most important thing! It's… for me you don't get that from other approaches, in other alternative medicines or other… even in allopathic medicine. You don't have that… I often say, making a good diagnosis, for me it's not a simple thing. Or, I want to say… it's taken me years to get to the point where I can figure that out a bit… But even now, I sometimes call the vet to get a diagnosis. Not necessarily for the treatment. And I find that the approach to diagnosis using homeopathy, for me, is incredibly important. And incredibly valuable, too… even more than all the remedies, really*.

(Interview with a farmer in the Manche, January 2015)

This was also the case with two female farmers in Normandy who practiced aromatherapy exclusively. One of them emphasized how she observes her animals more since she received training in this approach:

[The farmer is describing what she learned in the courses on aromatherapy:]

*Well, I would say, it gave us… the different things to observe when looking at the animal. (…) The eyes… the discharge from the nose, the chest… really everything you can observe but…. And then from there, it's like you apply it differently. And then, because of that, you are much more aware. So you observe much more*.

(Interview with a farmer in Normandy, March 2017)

This shift in how the farmer observes his or her animals leads in turn to a change in the farmers' relationship with their animals, how they work with the cows, bringing to the fore the sensory aspects of their daily work. Gaining these new observational skills so as to be able to detect health problems early thus emerges as a new element enabling the farmers to improve their overall management of herd health. Importantly, it is also a skill that allows them to access advisory services remotely, as we have seen: during a telephone consultation to help determine a homeopathic remedy, for example, the farmer can precisely describe the sick animal's condition and any changes in its behavior.

## Discussion

### Reflections on the Study Methodology and Characteristics of the Farmers Interviewed

Our study sample is not representative of French dairy farmers overall because our research was not designed to elucidate the opinions held by French dairy farmers in general with respect to the use of alternative veterinary medicines. Rather, our objective was to understand the perspective and experience of farmers already using homeopathy, phytotherapy and aromatherapy on their farms. Our key criterion in selecting farmers for the sample was to access a diverse range of uses of alternative medicines. For each field study area, the first step was to identify a group of farmers making regular or occasional use of homeopathy, phytotherapy or aromatherapy to treat herd health issues. To do so, we approached a number of actors connected to the local dairy sector, including advisory services, training organizations, and (in the case of Grand Ouest) veterinary practitioners. Given this approach, most of the farmers we interviewed had participated in activities associated with these organizations. This could be considered as a bias of our study, and represents one limitation of this type of qualitative approach, which necessarily involves using intermediaries to identify potential interviewees. To minimize this bias, we have drawn on different types of intermediaries, with the use of veterinarians in the Grand Ouest. Another potential strategy would have been to contact a company manufacturing and selling products typically used in veterinary phytotherapy, aromatherapy, or homeopathy (assuming the company would be willing and able to share their customers' information).

So this study does not enable us to describe the typical profile(s) of farmers, or farm operations, making use of alternative medicines. Nevertheless, there are elements of these farms' characteristics that stand out from our research. First, we found that the use of alternative medicines is not limited to the organic dairy sector. Second, for conventional farmers as for organic farmers, interest in these medicines goes hand in hand with a desire to reduce AMU and move toward a more holistic approach to herd health. Third, we identified a clear gender aspect to usage of alternative medicines, which we explore in the next section.

### Reducing Antibiotic Use on Dairy Farms: the Role of Women Farmers

A second feature that appears in the study sample is the role of women in the adoption of alternative medicines. A substantial number of women farmers were interviewed, either alone or together with their partners. Indeed, on the farms of our study sample it was frequently the women who had sought training in alternative medicines and/or had acquired the most expertise.

Research on the role of women in European agriculture has frequently emphasized the ways in which women are subject to forms of domination: although women have always played an active role in the work of agricultural production, their contributions have often been minimized, for instance by being lumped together with domestic chores (31). Women have thus been slow to gain official recognition of their professional work as farmers. The most frequently studied forms of emancipation for women in agriculture are (1) holding an off-farm job (32), and (2) the development of complementary on-farm enterprises, such as agri-tourism, direct sales of farm products, or small production enterprises (33, 34). Nevertheless, these activities of women farmers are often implicitly analyzed as external or peripheral to the farm's primary economic focus, thus confining women to a position of supporting their husband's work (35). These forms of women's entrepreneurial activity also receive weaker public policy support than those typically developed by men (34).

At a larger level, the increased specialization of farm operations and the ongoing professionalization of farming has coincided with a withdrawal of women from activities directly linked to the agricultural production of the farm. These trends are reinforced by mechanization, which has often entailed a replacement of women's work by machines (36). The role of women in agriculture thus appears to lie either outside of or on the periphery of the farm's primary production activities, or be limited to domestic tasks. By contrast, our study shows that women can occupy key positions within the farm's primary agricultural enterprise, including initiating new practices for livestock management. The domain of care, historically and culturally considered as belonging to women (37), is the domain where changes are first introduced—changes that then spread outward to other aspects of herd management. This observation calls for further research more specifically focused on women's role in the technical aspects of dairy management and in the adoption of new practices.

### Development of Observational Skills as a Strategy for Reducing On-Farm Antimicrobial Use

Several studies on the on-farm use of alternative medicines highlight farmers' “incorrect” use of these medicines. With respect to homeopathy, in particular, farmers are said not to perform a sufficiently thorough diagnosis of the animal's state of health, and to have a tendency to simplify the homeopathic approach by linking a given remedy with a given illness (38, 39). The “unicist” homeopathic approach—the prevailing approach in the world of veterinary practice—holds that every sick individual is affected in a unique way by a given disease, and thus requires a specific, unique treatment. In our interviews, farmers expressed their challenges in adhering to the unicist principle, which they found complicated and requiring many years of study. Nevertheless, they found the use of homeopathic medicine to have practical value for their farms, enabling them to better care for their animals.

How can we understand the fact that farmers find these medicines effective, while some authors argue that they don't use them correctly? Our results make it possible to move beyond this apparent dilemma, showing how the use of alternative medicines fits into overall dairy farm management and supports a holistic approach to herd health. Science and the veterinary profession see an opposition between conventional medicine and alternative medicine; but dairy farmers use both in a practical fashion, simultaneously or sequentially, with the underlying goal of better managing risks to herd health. Hektoen (24) found similar results in a study of Norwegian dairy farmers, who likewise view homeopathy as a new tool, among other tools, to be used in caring for their cows. As noted previously, moreover, the instructors of these training courses in alternative medicines present close observation of the animals as a central topic. In the training course we attended, the instructor in aromatherapy repeatedly emphasized the importance of closely and regularly observing the animals' condition, offering a series of charts for use in assessing specific health problems—charts we later observed farmers making use of. Similarly, close, careful observation of the animal is central to the homeopathic approach to veterinary care. Clinical diagnosis in homeopathy is based on a large number of precise visual indicators relating to the condition of the animal's body, specific aspects of its behavior, and characteristics of its excreta. To perceive changes in the behavior of a given animal, one must be in the habit of regularly and closely observing the herd. All of this suggests that the efficacy farmers experienced in alternative medicines is related to the larger effect of the whole approach to care they adopt when they use these medicines, including closer attention to the herd.

Acquiring skills in the direct observation of livestock requires changing how one works with the herd, making it possible to reprioritize the sensory dimension of the farmer's relationship with his or her animals. Observational skills are not ordinarily taught in agricultural schools, however. They are generally considered to be innate, or as a form of practical knowledge passed from father to son, or from employer to student during farm apprenticeships (40, 41). Nevertheless, our results show this type of practical know-how, based on sensory elements, can be effectively formalized and in this way taught to farmers. The training courses offered to farmers in connection with alternative medicines thus constitute one pathway, among others,^12^ for developing farmers' observational skills.

In scientific literature, farmers training are considered as a main driver for AMU reduction (6, 43). The challenge is to improve farmers knowledge regarding use of antibiotics and prevention methods. Our results show light on another category of skills: the observational skills, that are of importance when farmers aim at improving animal health management.

### Alternative Medicines Suggest New Ways for Veterinarians to Work With Farmers

Our results also have relevance for ongoing discussions with regard to the changing role of veterinarians on dairy farms. The fight against AMR—and more generally the increased demand among citizens and consumers for better management of livestock health and a greater respect for farm animal welfare—hold consequences for the veterinary profession: often regarded simply as providers of urgent care or as intermediaries for the delivery of veterinary pharmaceuticals, veterinarians are now being asked to place more emphasis on advisory services and preventive medicine (8, 44). As Fortané et al. (45) have shown for the pig farming sector, reducing on-farm antibiotic use requires changing the nature of the relationship between the farmer and his or her professional network, of which veterinarians are an important part.

The farmers we interviewed have invested time and money in improving their animal health management practices. These farmers have turned to short training courses offered by agriculture-related organizations in part because of the lack of specialists in alternative medicines in their local professional milieus. Most rural veterinarians have little interest in seeking training in forms of medicine whose efficacy has not scientifically established (21, 46). As we have seen, however, demand for alternative medicines exists not only among organic dairy farmers but also among dairy farmers more generally. What is more, this demand is indicative of broader changes in farmers' needs and expectations with respect to managing the health of their animals: a desire to focus more prevention, a desire to adopt a more holistic approach (47, 48). A better understanding of farmers' interest in homeopathy, aromatherapy, and phytotherapy—and more importantly, an understanding of the broader needs and expectations underlying that interest—can provide veterinarians with useful information for rethinking their professional interactions with farmers.

In the scientific literature to date, the primary avenues that have been explored with regard to the future role of veterinarians involve the creation of HACCP-style management systems such as Veterinary Herd Health Management (VHHM) (9) and Animal Health and Welfare Planning (AHWP) (49). With VHHM and AHWP, the veterinarian develops a herd health management plan based on explicit objectives defined in consultation with the farmer, and then conducts regular assessments to see how the farm is doing with respect to the plan (49, 50). A variety of challenges can emerge with this type of initiative, however: objectives are not always clearly established; and it can be difficult to measure the extent to which the farmer has followed the veterinarian's advice, or the effects of specific recommendations on herd health (51). More fundamentally, some farmers are reluctant to participate in such initiatives. Jansen et al. (52), after conducting a study to identify the causes of this reluctance, emphasized the manner in which the veterinarian communicates with the farmer: the former must tailor his or her language to fit the latter's way of thinking about herd health management. To work effectively as advisors, veterinarians also need to improve their listening skills, seeking to understand the farmer's perspective—the logic underlying his or her practical decision-making (53). For Duval et al. (21), too, veterinarians must be proactive, making the most of their conversations with farmers to suggest improvements in herd health management.

Finally, developing an advisory role for veterinarians implies rethinking the economic model of the veterinary profession since in many countries, veterinarians both prescribe and sell veterinary pharmaceuticals. For example, in France, nearly 70% of rural veterinarians' income comes from the sale of medicines (54). Restrictions on the sale of antibiotics by veterinarians is seen as a way to reduce AMU, but could lead to a significant reduction in revenue for rural veterinary practices (8, 54) and thus contribute to the loss in numbers of rural veterinary services (44). Our study, however, has identified potential strategies for how the advisory services of veterinarians could be remunerated. Furthermore, we identified different types of professional connections between farmers and alternative medicine specialists, corresponding to different types of paid services: short training courses for farmers; periodic phone consultations; annual farm visits to conduct an overall review of herd health management practices. To this may be added the farmer-to-farmer discussion groups organized by some homeopathic veterinarians (55, 56). These groups are similar to the “stable schools” organized in Denmark (57), in which a group of livestock farmers meet at one of their homes, defining problems to be discussed in advance and sharing their experiences of specific treatment failures and successes. Skilled facilitators are essential to the smooth functioning of such groups (58).

A study of organic dairy farmers' advisory networks for animal health issues (59) showed different forms of annual contracts established between farmers and veterinarians. Some are proposed specifically to farmers converting to organic, to help them during the conversion period. In sparsely populated areas with few practicing veterinarians, groups of farmers have created a system of annual contracts with one or more veterinarians to ensure that veterinary services remain available. In this case, the contracts can cover a variety of services, including farm visits, telephone consultations, trainings, and the facilitation of farmer-to-farmer discussion groups. Like VHHM and AHWP, these annual contracts are based on the idea of regular monitoring of the herd by the veterinarian, but they are more flexible because it is the farmer who determines the frequency of the consultations, according to his or her needs. In addition, these kinds of activities require different skills on the part of the veterinarian, including a different approach to interacting with farmers. Service contracts with farmers thus appear to be a useful strategy by which veterinarians can be remunerated for advisory services, albeit one that places them in direct competition with other dairy services professionals.

## Conclusion

Although their effectiveness is controversial, alternative medicines are currently considered to be one strategy among others for reducing antibiotic use in livestock agriculture. Alternative medicines are also in regular and widespread use by both organic and conventional dairy farmers. In this article, we sought to take dairy farmers' interest in homeopathy, aromatherapy, and phytotherapy seriously, studying in detail their use of these therapies for herd health management. We found that alternative medicines are not understood by farmers as a substitute for conventional medicine; rather, these medicines play a role in a holistic approach to herd health that includes both preventive measures and a variety of curative treatments, grounded in careful and continuous observation of the animals' state of health. Farmers employ criteria for the observation and interpretation of animals' condition that are fundamental to veterinary homeopathy and aromatherapy. Although short training courses are the primary avenue by which farmers learn about alternative medicines, individual advisory relationships with alternative medicine specialists can follow on from these courses. Farmers' interest in alternative medicines thus suggests larger expectations and needs for advisory services and assistance with respect to the integrated management of animal health. Understanding these needs and expectations offers useful avenues for rethinking the place of veterinarians on dairy farms: to move beyond the role of “prescriber of medicines” and more toward that of a farming advisor. This involves more than simply educating farmers as to good practices for antibiotic use and disease prevention; it should also mean helping farmers develop their skills for monitoring herd health and appropriately treating animal health issues.

## Data Availability Statement

The data analyzed in this study is subject to the following licenses/restrictions: Interviews with farmers are confidential data. Requests to access these datasets should be directed to florence.hellec@inrae.frandlt.

## Ethics Statement

Ethical review and approval was not required for the study on human participants in accordance with the local legislation and institutional requirements. Written informed consent for participation was not required for this study in accordance with the national legislation and the institutional requirements.

## Author Contributions

FH, CM, and MJ participated directly in the collection and analysis of the empirical data. FH and CM jointly conducted the field survey in Franche-Comté and developed the analytical grid for the data collected in Franche-Comté and Normandy. FH conducted the field survey in Normandy. MJ made the field survey in Grand Ouest. FH and MJ used this grid to analyse data collected in Grand Ouest. FH wrote an initial version of this article. CM and MJ read and commented this version. All authors approved the final version of this article.

## Conflict of Interest

The authors declare that the research was conducted in the absence of any commercial or financial relationships that could be construed as a potential conflict of interest.

## Footnotes

^1^The French action plans implemented since 2012 to fight against AMR in veterinary medicine are presented on the website of the French ministry of agriculture (https://agriculture.gouv.fr).

^2^Homeopathy is a therapeutic method developed by Samuel Hahnemann from the end of the eighteenth century. It is based on the so-called “doctrine of similars”–the idea that any disease may be treated with dilute preparations of substances that would ordinarily (in healthy individuals, in undiluted form) produce symptoms similar to those associated with the disease. Most homeopathic remedies are based on plant extracts, although some are made from animal products. Homeopathic medicines are typically administered in the form of granules or liquids.

^3^Phytotherapy seeks to treat or prevent diseases using medicinal plants (whole plants or plant parts, dried or fresh). Aromatherapy is based on the use of plant extracts in the form of essential oils.

^4^Most phytotherapy and aromatherapy products are not considered medicines under French law and are thus not authorized for use for therapeutic purposes. They are usually marketed as dietary supplements by the companies that make and sell them. Homeopathy occupies a more ambiguous position under French law: homeopathic remedies may be sold as medicines but are not regulated for veterinary use. Livestock farmers in France typically purchase homeopathic remedies from pharmacies; most veterinary practices offer few if any homeopathic products for sale.

^5^The first and second authors on this paper completed the field work in Franche-Comté as a team; the first author alone conducted the field work in Normandy.

^6^For example, some dairy farmers who were using homeopathy sought to explain to us in detail their methods for observing sick animals, showing us the kinds of notes they make and giving specific examples.

^7^The third author on this paper conducted the interviews in the Grand Ouest with the assistance of a homeopathic veterinarian. The idea for writing this article emerged from an informal discussion among the three authors: the first and second authors proposed to the third the idea of pooling and comparing the three datasets, applying the analytical grid developed in the first two studies to the data from the third.

^8^The questionnaire included detailed questions relating to herd management (preventive measures such as hygiene and careful monitoring of the herd, choice of allopathic and homeopathic treatments for the most common herd health issues) followed by a series of statements relating to the risks of antibiotic resistance and the value of homeopathy for addressing these risks (farmers were asked to indicate if they were more or less in agreement with these statements). Data from this portion of the third study are not addressed in this article since we had no analogous data from the other two studies.

^9^MWU is a measurement of work hours in agriculture: one MWU corresponds to one person working full-time on the farm operation.

^10^*A PPE (plan prévisionnel d'exploitation, or future farm plan) is established when a farmer receives from federal subsidies to begin farming. The PPE can specify various obligations on the part of the farmer, including taking additional training courses*.

^11^Developed by Dr. Bruno Giboudeau in the early 2000s, the OBSALIM® method seeks to detect dietary imbalances using specific criteria for the observation of individual animals and/or the whole herd. These criteria are directly inspired by the observational approach used in homeopathy (30).

^12^Training courses in the Cows Signal® approach, based exclusively on animal observation, are also offered in many European countries (42).
